# Effect of humic acid on ciprofloxacin removal by magnetic multifunctional resins

**DOI:** 10.1038/srep30331

**Published:** 2016-07-28

**Authors:** Wei Wang, Jiade Cheng, Jing Jin, Qing Zhou, Yan Ma, Qingqing Zhao, Aimin Li

**Affiliations:** 1 State Key Laboratory of Pollution Control and Resource Reuse, School of the Environment, Nanjing University, Nanjing 210023, P. R. China

## Abstract

Background organic matter significantly influences the removal of emerging contaminants in natural water. In this work, the adsorption of ciprofloxacin (CPX) onto a series of magnetic multifunctional resins (GMA10-GMA90) in the presence and absence of humic acid (HA) was conducted to demonstrate the effect of HA. Both hydrophobic and ion exchange interactions contributed to CPX adsorption. Negative charge-assisted hydrogen bonds also participated in the adsorption process, resulting in the high adsorption amount of anionic CPX onto the negatively charged GMA30 under basic solutions. HA could impact CPX adsorption not only as a competitive adsorbate but also as an additional adsorbent. At pH 5.6, the additional adsorption sites provided by adsorbed HA molecules on the resins dominated and thus facilitated the adsorption process. While at pH 10, HA inhibited the adsorption of CPX by directly competing for ion exchange sites and coexisting with CPX in the solution. The ratio of the amount of CPX adsorbed by dissolved HA to that by the resin reached as high as 1.61 for GMA90. The adsorbed HA molecules onto the resins could provide additional adsorption sites for CPX as proven by the enhanced CPX adsorption in HA-preloading systems at pH 5.6.

Magnetic resins are widely applied as a new type of adsorbent in water treatment because of their convenient magnetic separation property and superior regeneration performance^1^,^2^,^3^,^4^. MIEX^®^ produced by Orica Watercare, Australia is currently the most widely used magnetic resin. MIEX^®^ efficiently eliminates natural organic matter (NOM) through its quaternary amine functional groups^5^,^6^,^7^,^8^. NOM is the precursor of disinfection by-products, and the efficient removal of NOM can greatly improve the quality of drinking water^9^,^10^. Thus, magnetic resins serve as suitable application prospects, and multiple water treatment projects have been established worldwide.

Considering the suitability of magnetic resins, researchers have attempted to utilize these materials to eliminate emerging contaminants (ECs), including pesticides, pharmaceuticals and personal care products, and endocrine-disrupting compounds, from aquatic environments. Liu *et al*. observed that MIEX^®^ can efficiently remove bentazone in 30 min at the initial concentrations of 0.04–0.12 mmol/L^11^. Neal *et al*. found that the MIEX^®^ resin can remove approximately 70% of predominantly negatively charged estrone^12^. However, the MIEX^®^ resin essentially removes organics by ion exchange; hence, the neutral form of most ECs under natural conditions inhibits the application of MIEX^®^ for ECs removal^13^,^14^.

To overcome this limitation, our group developed novel hypercrosslinked magnetic resins that display high affinity to neutral pollutants^15^,^16^,^17^. The high specific surface area and abundant micropores of hypercrosslinked magnetic resins facilitate the adsorption of hydrophobic organic contaminants. However, hypercrosslinked resins exhibit a poor removal efficiency for hydrophilic organic contaminants. Other studies prepared multifunctional resins that contain both ion exchange groups and possess high specific areas, and found that these resins display favorable adsorption capacities for both hydrophilic and hydrophobic pollutants^18^,^19^. Thus, these resins were considered promising adsorbents for ECs removal.

Background NOM, which comprises a complex mixture of organic molecules that vary in size and chemical structure, is ubiquitous in drinking water sources and seriously affects ECs purification by adsorbents. As the representative NOM, humic acid (HA), is found to have a great impact on the adsorption of ECs. Previous studies confirmed that HA can compete with ECs for adsorption sites and block the pores on the adsorbents^20^,^21^. Meanwhile, Aristilde *et al*. found that a complex interaction exists between ECs and HA^22^,^23^, which could influence the fate of ECs and thus affect their adsorption. In addition, the interaction between HA and resin was found to be diversiform^24^. Thus, the adsorption mechanisms of ECs onto multifunctional resins in the presence of HA must be further studied. The effect of the content of ion exchange groups on ECs adsorption in the presence of HA also requires investigation.

In this work, a series of multifunctional resins with different amounts of ion exchange groups were synthesized via a sequence of polymerization, amination and post-crosslinking reactions. As one of the widely used drugs in human and veterinary therapeutic treatment^25^, ciprofloxacin (CPX), which can induce antibiotic resistance genes in microorganisms and pose health risks to human health^26^, is selected as target compound. As an amphoteric substance, CPX molecules represent different electric properties at diverse pH. The adsorption of CPX in different forms could enhance the understanding of the adsorption behaviors of ECs with different properties. Meanwhile, the adsorption of CPX in the absence and presence of HA was compared to assess the interaction mechanisms between CPX and HA on the resins.

## Materials and Methods

### Materials

Divinylbenzene (DVB) (80 wt%), glycidyl methacrylate (GMA) (97 wt%), CPX, and HA were obtained from J&K Chemical Co. Ltd. (China). Benzoperoxide, ferric chloride, ferric chloride hexahydrate, ferrous chloride tetrahydrate, acetone, aqueous ammonia (25 wt%), oleic acid, methanol, ethanol, toluene, gelatin, trisodium phosphate, disodium hydrogen phosphate, sodium hydroxide, sodium chloride, hydrochloric acid, and 1,2-dichloroethane were of analytical grade and purchased from Sinopharm Chemical Reagent Co. Ltd. (China). Dimethylamine was produced by Shanghai Chemical Reagent Corp. (China). Five magnetic multifunctional resins designated as GMA10 to GMA90 were obtained through a sequence of polymerization, amination, and post-crosslinking reactions. Detailed procedures for the preparation of resins are described in the Supplementary Information.

### Characterization of the multifunctional resins

Fourier-transform infrared spectrometry (FTIR, Nexus870, Nicolet, USA) was employed to identify the surface functional groups. The surface properties and specific surface area of the resins were determined by conducting nitrogen adsorption–desorption experiments at 77 K and by using the standard Brunauer–Emmett–Teller (BET) equation, respectively. The zeta potentials of the resins were measured by using a JS9H-type electrophoresis apparatus (Shanghai Zhongchen Digital Technology Equipment, China).

### Batch adsorption experiments

For single adsorbate adsorption experiments, 100 mL of solutions with an initial concentration of 0.03 mmol/L CPX or 20 mg/L HA were shaken at 150 rpm and 293 K with 0.01 g of each resin in 250 mL conical flasks. Initial solution pH was adjusted using 0.1 mol/L HCl and 0.1 mol/L NaOH.

Adsorption isotherms were conducted under two pH conditions (pH 5.6 and 10) to evaluate the adsorption mechanism. Each isotherm was divided into three types. For single-solute systems, 0.01 g of resin was introduced into a series of 250 mL conical flasks with 100 mL of CPX solutions at different initial concentrations. For HA–CPX competitive adsorption experiments, 0.01 g of resin was introduced into a series of 250 mL conical flasks with 100 mL of mixed solutions. The mixed solutions were prepared in advance by mixing HA and CPX solutions. The concentration of the CPX solutions remained the same as those in the single-solute experiments, and the concentration of the HA in the solution was 20 mg/L. For HA preloading followed by CPX adsorption experiments, 0.01 g of resin was first contacted with a 20 mg/L HA solution in a series of 250 mL conical flasks for 24 h. Afterward, a series of 100 mL CPX solutions was mixed with the HA preloaded resins in the other conical flasks. The equilibrium adsorption time for CPX was 2 h. All of the experiments were repeated thrice to obtain average values. The amount of CPX adsorbed per unit mass was determined using the following equation:


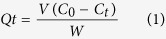


where *C*_0_ and *C*_t_ represent the initial concentration and concentration at time t (min), respectively (mmol/L); *V* (L) is the volume of the aqueous phase; W (g) is the amount of resin used; and *Qt* (mmol/g) is the adsorption capacity at time *t*.

### Determination of CPX

Prior to CPX analysis, the pH of all the solutions was adjusted to 2, and the CPX in the HA–CPX competitive adsorption experiments was measured also at the original pH. High-performance liquid chromatography (Agilent 1200) with an Eclipse XDB-C18 column (4.6 × 250 mm, 5 μm; Agilent). The mobile phase comprised 80% acetonitrile and 20% water (with 0.1% formic acid, w/w) at a flow rate of 1 mL·min^−1^. The DAD detection wavelength was 276 nm, and the column temperature was 303 K. The retention time for CPX was 4.06 min and the detection limit was 0.1 μmol/L.

### Data Analysis

To obtain an enhanced understanding of the adsorption mechanism, three isotherm models were employed to fit the adsorption data^27^,^28^:


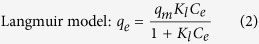



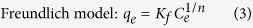



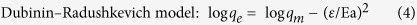


where *q*_*e*_ (mmol/g) is the equilibrium adsorption capacity, *q*_*m*_ (mmol/g) represents the maximum monolayer adsorption amount, *K*_*l*_ (L/mmol) is the Langmuir adsorption affinity parameter, and *K*_*f*_ and *n* are Freundlich adsorption constants. Furthermore, ε (kJ/mol) = −RT ln (1 + 1/C_e_) is the effective adsorption potential, where R is the universal gas constant (8.314 × 10^−3^ kJ/mol∙K) and T is the absolute temperature (K). Ea denotes the Dubinin–Radushkevich fitting parameters (energy of adsorption).

## Results and Discussion

### Characterization of the multifunctional resins

**Figure 1:**
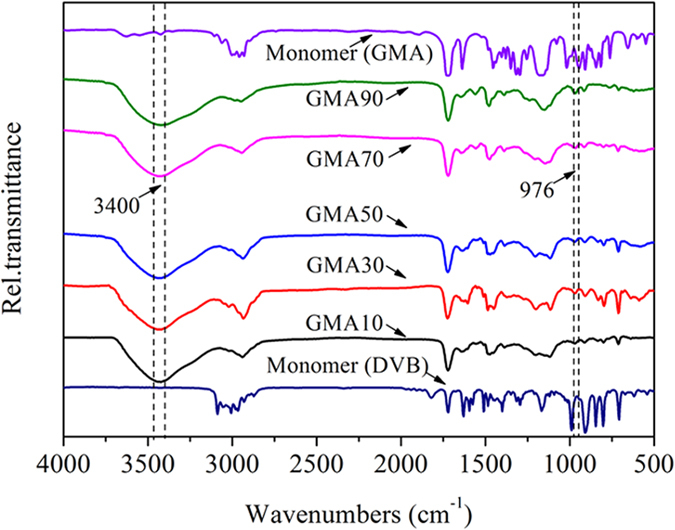
FTIR spectra of the multifunctional resins and the monomers.

Five magnetic multifunctional resins designated as GMA10 to GMA90 were obtained via amination and post-crosslinking reactions of the magnetic poly (DVB-co-GMA) beads which were prepared from varied GMA dose. Figure 1 depicts the FTIR spectra of the resultant beads. Compared with the copolymerization monomers, the absorbance peaks at ~976 cm^−1^ and ~3400 cm^−1^ of the resins correspond to the C–N stretching vibration and the O–H in-plane bending vibration, respectively, which confirms the successful introduction of the –N^+^(CH_3_)_3_ group after amination^29^,^30^. The physicochemical properties of these resins are listed in Table 1. GMA10 possesses the largest BET surface area (S_BET_; 1109.25 m^2^/g) and the lowest total anion exchange capacity (TEC; 0.92 mmol/g). The S_BET_ value of the resins sharply increased from 1.17 m^2^/g to 1109.25 m^2^/g when the content of DVB increased from 10% to 90%. A positive correlation was observed between S_BET_ and DVB content, with a high correlation efficient (R^2^) value of 0.915 (Figure S1A). A positive correlation was also noted between TEC and GMA content (Figure S1B). The TEC value significantly increased from 0.92 mmol/g in GMA10 to 3.71 mmol/g in GMA90 with increasing GMA content. The results indicated that resins with different properties can be obtained by adjusting the ratio of DVB and GMA.

### Adsorption performance: effect of pH

**Figure 2:**
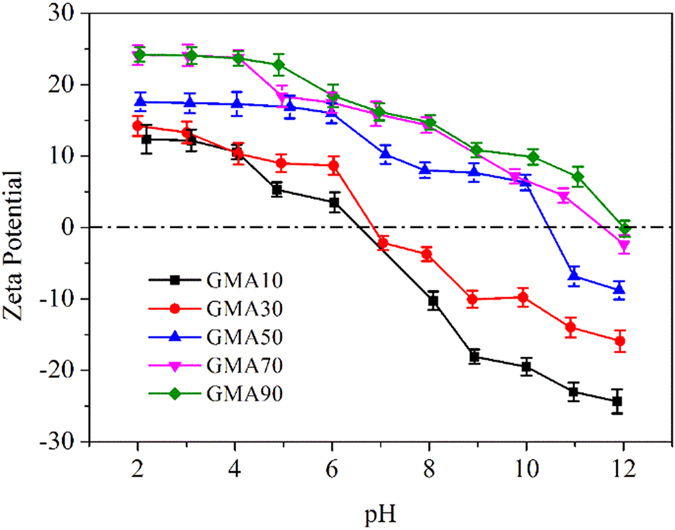
Zeta potentials of resins.

The TEC values of the resins indicated that the resins differed in surface charge. For a given resin, the surface charge is pH dependent. With pH change, the zeta potential of each resin varied and consequently affected the adsorption. As displayed in Fig. 2, the zeta potentials of all the resins were positive at low pH and then gradually became negative with increasing pH. The zero potential point of the five resins increased with increasing GMA content. Meanwhile, CPX as an amphoteric substance has two pKa values and may exist as three species (i.e., CPX^+^, CPX^+−^, and CPX^−^) at different pH levels (Figure S2). Thus, exploring the effect of solution pH on CPX adsorption is highly important.

**Figure 3:**
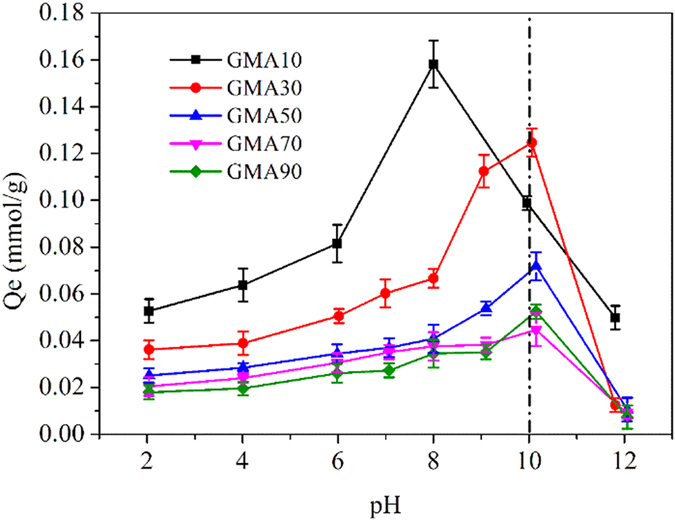
Effect of solution pH on CPX adsorption (C_CPX_ = 0.03 mmol/L, 0.01 g of adsorbents, temperature = 293 K).

Figure 3 shows the influence of solution pH ranging from 2 to 12 on CPX adsorption. The adsorption amount of CPX onto all the five resins initially increased with pH but decreased at high pH ranges. For GMA10, the maximum adsorption of CPX occurred at pH 8 when CPX was zwitterion. The hydrophobic interaction and π–π bonding dominated under this condition because of the high surface area of GMA10^16^,^31^. As to the four other resins, the maximum adsorption capacity reached at pH 10. CPX was negatively charged, whereas GMA50, GMA70, and GMA90 remained positively charged. The electrostatic interaction caused by the opposite charge facilitated CPX adsorption. However, the adsorption of CPX on GMA30 followed different patterns from those of the other adsorbents. Both GMA30 and CPX were negatively charged at the pH range of 8–10. However, this occurrence did not reduce CPX uptake by electrostatic repulsion. Instead, the adsorbed amount of CPX increased and peaked at pH 10. The abnormal result may be caused by the negative charge-assisted hydrogen bonds ((−)CAHBs)^32^,^33^. The absolute difference in pKa (ΔpKa) between the pKa of CPX (6.48, proton donor group) and the point of zero charge of GMA30 (6.6, proton acceptor group) was small, which led to the formation of strong (−)CAHBs^34^. At pH > 10, the electrostatic repulsion between the negatively charged resins and CPX^−^ restricted the adsorption of all the resins.

Compared with the five resins, the adsorption amount of CPX increased with increasing DVB content at low pH, which suggested that the hydrophobic interaction dominated. At pH 10, CPX^−^ dominated, and ionic forces played a great role in CPX adsorption. Consequently, the adsorption amount of CPX onto the resins with high TEC increased. The maximum adsorption amount onto GMA90 at pH 10 was even higher than that onto GMA70, which may be attributed to the dominance of ion exchange in CPX adsorption for GMA90. Despite the similar electric charges for the GMA50 and GMA70 at pH 10, the stronger hydrophobic interaction and π–π bonding between GMA50 and CPX caused by higher BET surface area led to the higher adsorption amount of GMA50. Furthermore, the adsorption of CPX onto GMA30 caused by (−)CAHBs was the highest among the tested resins, demonstrating that (−)CAHBs played an important role in adsorption.

### Adsorption performance: effect of HA

**Figure 4:**
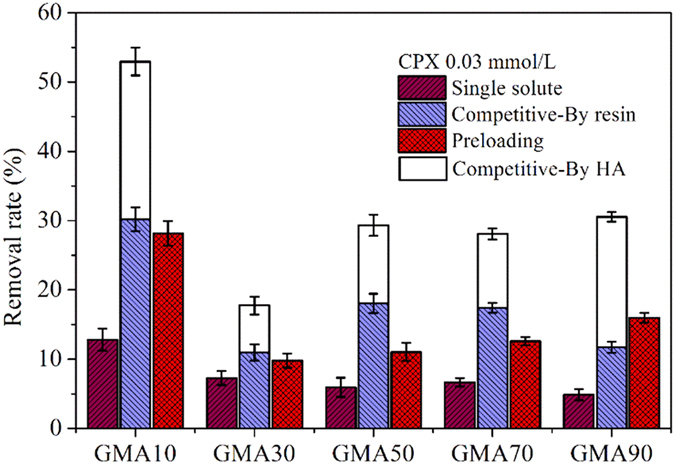
Removal rate of CPX by the resins in the absence and presence of HA (pH = 5.6, C_CPX_ = 0.03 mmol/L, C_HA_ = 20 mg/L, 0.01 g of adsorbents, temperature = 293 K).

In natural water, CPX does not exist alone but with HA, and the absence of HA could considerably affect CPX removal. Previous studies showed that HA affects the adsorption of micropollutants onto activated carbon by direct competition for adsorption sites^35^,^36^. Apart from physical adsorption sites, ion exchange groups were also present in the multifunctional resins. The mechanism was complex for the adsorption of micropollutants onto resins in the presence of HA. The removal of CPX in the absence and presence of 20 mg/L HA by the resins at pH 5.6 is shown in Fig. 4. The fractions of CPX were 87.11% for CPX^+^, 12.88% for CPX^+−^, and 0.01% for CPX^−^ (Figure S2). The removal rate of CPX by GMA10 was 12.82%, which is extremely close to the proportion of CPX^+−^. This result confirms the physical adsorption of CPX onto GMA10 and the lack of significant contribution of electrostatic interaction at this pH. The four other resins showed lower removal rates because of their decreased surface areas that weakened CPX adsorption.

In the presence of HA, the interaction between the positively charged CPX and the negatively charged HA was not negligible (Figure S3). The additional HA could decrease the peak area under this condition; thus, the removed CPX could be divided into two parts: (i) one that interacted with HA in the solution and (ii) the other that was removed by the resins. The ratio of the amount of CPX adsorbed by HA in the solution to that by the resins showed a minimal difference from GMA10 to GMA70 (0.61–0.75), except for that in GMA90 (1.61). For GMA90, the positively charged surface hold back positively charged CPX^+^ and lower S_BET_ could not ensure the adsorption amount for CPX^+−^. Conversely, the electrostatic interaction between CPX and HA was stronger, and the CPX amount that interacted with HA in the solution was even higher than that removed by the resins.

Moreover, the amount of CPX removed by the resins in the presence of HA was much higher than that in the absence of HA. This finding indicates that HA acted not only as an additional adsorbent for CPX but also as a “bridge” between the resins and CPX. The negatively charged HA was adsorbed onto the positively charged resins and reduced the electrostatic repulsion between the resins and CPX^+^, which benefited CPX adsorption. In addition, the adsorbed HA onto the resins could provide additional adsorption sites for CPX and enhance CPX adsorption onto the resins.

When the resins were preloaded with HA, the removal rate of CPX by GMA10 to GMA70 was lower than that in the binary solutions but much higher than that in pure CPX (Fig. 4). This occurrence was due to the fact that the adsorption sites were occupied by HA more seriously than in the binary solutions and much bridging action promoted adsorption process than in single solute. Moreover, the bridging action was much stronger than the adverse effect. By contrast, for GMA90, the removal rate of CPX onto the resins in the preloading system was the highest among those of the others resins. GMA90 exhibited the highest zeta potential and adsorbed most HA molecules (Figure S4). The preloaded HA molecules compensated for the decreased adsorption sites caused by the low S_BET_ and provided extra adsorption sites for CPX, which led to the high removal rate.

### Adsorption isotherms

**Figure 5:**
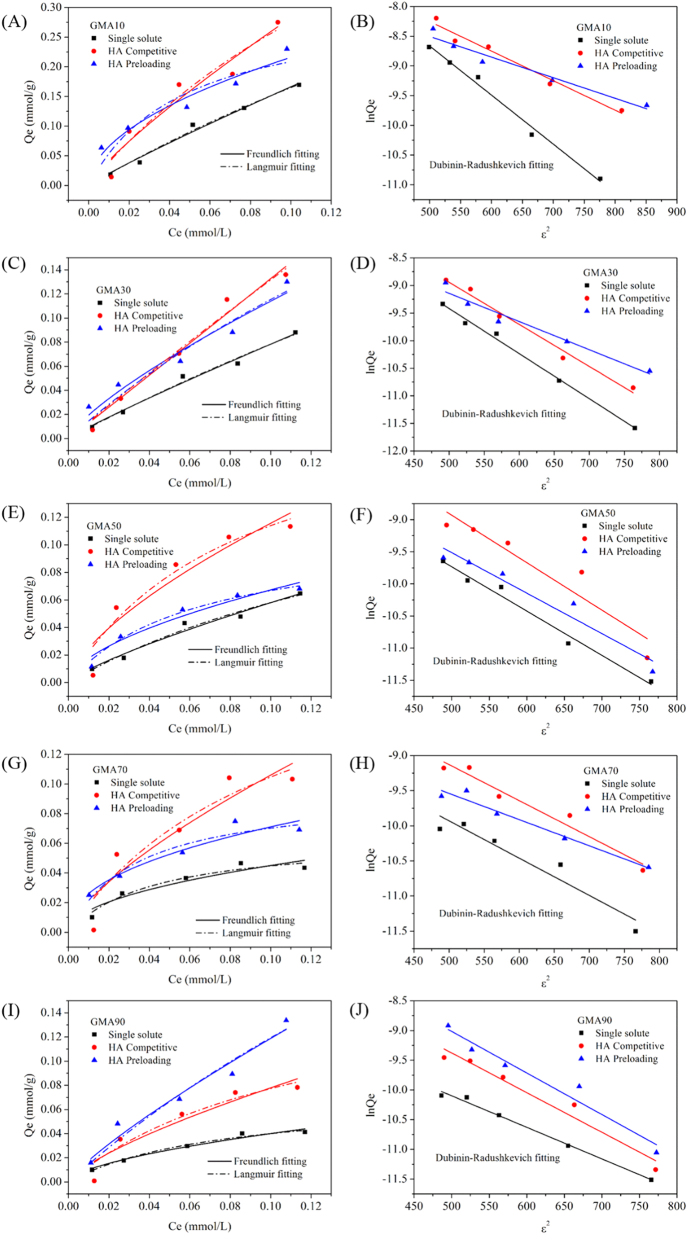
Adsorption isotherms of CPX onto (**A,B**) GMA10, (**C**,**D**) GMA30, (**E,F**) GMA50, (**G,H**) GMA70, and (**I**,**J**) GMA90 (pH = 5.6, temperature = 293 K).

**Figure 6:**
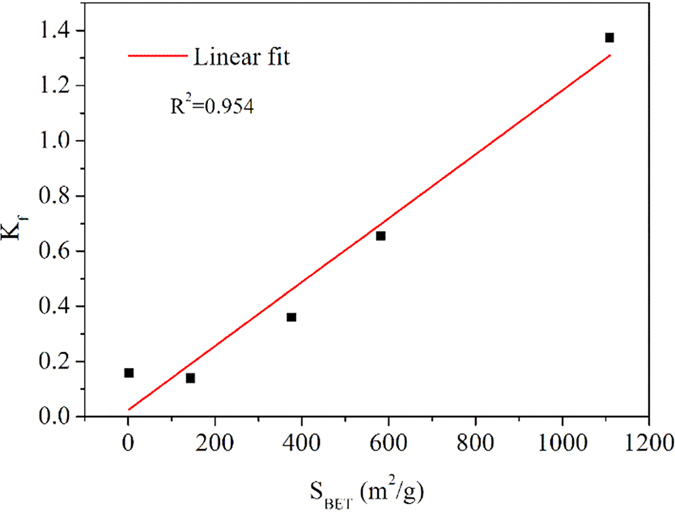
Linear relationship between the parameter of the Freundlich model (K_f_) and S_BET_.

To further explore the mechanism of how HA affected the adsorption of CPX onto multifunctional resins, the adsorption isotherm experiments at pH = 5.6 and 293 K were conducted and the results are displayed in Fig. 5. Table 2 summarizes the simulated parameters of the isotherm models. As shown by the correlation coefficients (R^2^), the adsorption of CPX onto the resins fitted well with both Freundlich and Langmuir models. In the absence of HA, the adsorption amount of CPX followed the order of GMA10 > GMA30 > GMA50 > GMA70 > GMA90, which was consistent with the order of the surface area of the resins. Thus, a linear regression analysis was conducted between the K_f_ values obtained from the Freundlich models and the S_BET_ values. As displayed in Fig. 6, a positive correlation was observed between the K_f_ values and the S_BET_ of all the resins. The adsorption of CPX onto the resins was primarily attributed to their available surface area because no other relationship was found between K_f_ and other structural parameters of the resins. All of the five resins showed better adsorption performance for CPX in the presence of HA, either in the binary solution or in the preloading system. Meanwhile, the preloaded GMA90 showed a higher affinity for CPX than that in the binary systems, whereas the four other resins exhibited the opposite effect. In the presence of HA, almost all of the K_f_ values were higher than those in the single CPX solutions. The adsorbed HA molecules could improve the affinity to CPX molecules and neutralize a part of the surface charge of the resins. Apart from GMA90, the values of K_f_ for the other four resins were higher in the binary systems than in the preloading systems, which could explain the higher affinity. By contrast, for GMA90, the higher amount of the adsorbed HA molecules in the preloading systems could shield the positive surface charge of the resin and provide more adsorption sites for CPX.

**Figure 7:**
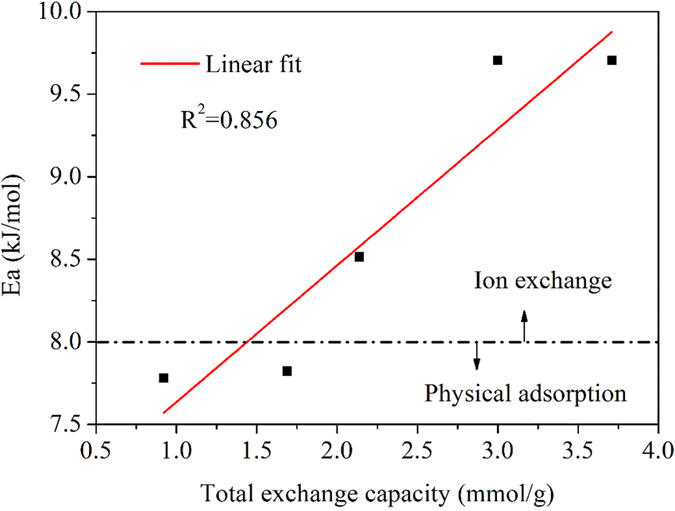
Linear relationship between adsorption energy (Ea) and TEC.

Adsorption energy is also an important parameter that reflects the adsorption mechanism. Thus, the Dubinin–Radushkevich model was also employed to simulate adsorption. The fitting results are listed in Table 2. Previous studies showed that the adsorption energy (Ea) between 1 and 8 kJ/mol signifies physical adsorption^37^, whereas an Ea range of 8–16 kJ/mol represents an ion exchange mechanism^38^. In our study, the Ea values for the adsorption of CPX onto GMA10 and GMA30 were 7.78 and 7.83 kJ/mol, respectively, which indicates that the physical adsorption dominated. The values of Ea increased with increasing TEC value, indicating that the ion exchange interaction became primary. A positive correlation was observed between the Ea values and the anion exchange capacity of all the resins (Fig. 7). Meanwhile, the increased values of Ea in the presence of HA indicate that HA enhanced the effect of ion exchange and thus enhanced the adsorption process.

The form of the adsorbates could affect the adsorption efficiency, as described above. Adsorption isotherm experiments at pH 10 were also conducted to investigate further how HA affects the adsorption of CPX onto the multifunctional resins, and the results are displayed in Figure S5. The simulated parameters of the isotherm models are summarized in Table S1. Different from the adsorption performance of CPX at pH 5.6, the adsorption amounts of CPX in the absence of HA were much higher than those in the presence of HA. The higher values of K_f_ in single CPX solutions could explain the disparity in the adsorption capacity. The adsorption amount of CPX onto GMA90 was also higher than that onto GMA70. As mentioned earlier, the high anion exchange capacity of GMA90 facilitated the attraction of negatively charged CPX molecules. Furthermore, HA could restrict CPX adsorption by direct competition for ion exchange sites, especially in preloading systems. The values of Ea decreased in the presence of HA, indicating the shift of the interaction mechanism from ion exchange to physical adsorption.

### Mechanism simulation

**Figure 8:**
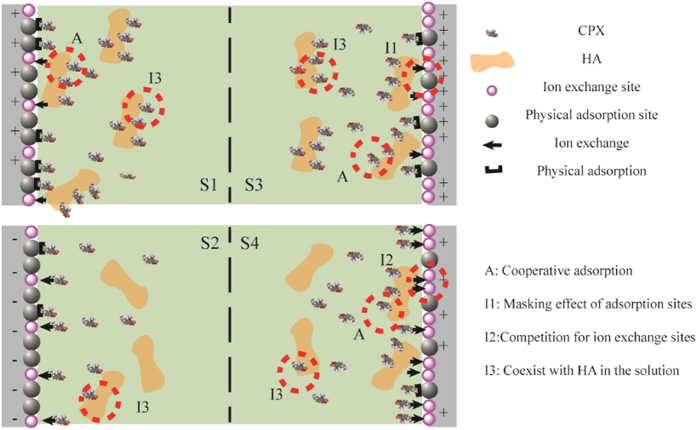
Schematic of the acceleration and inhibition mechanisms of the adsorption of CPX onto multifunctional resins (S1: Resins with high S_BET_ at pH 5.6; S2: Resins with a high S_BET_ at pH 10; S3: Resins with a high TEC at pH 5.6; and S4: Resins with a high TEC at pH 10).

These discussions suggested that the interaction among CPX, HA, and resins should be responsible for CPX adsorption onto multifunctional resins. In single solute systems at pH 5.6, CPX was primarily adsorbed by physical adsorption. Conversely, ion exchange played an important role when most of CPX was present in the form of CPX^−^ at pH 10. In the presence of HA, the adsorption of CPX onto the resins could be affected through enhancing effects, such as cooperative adsorption for CPX (A), and inhibitory effects, including the masking effect of adsorption sites (I1), direct competition for ion exchange sites (I2), and coexistence with HA in the solution (I3). The dominating effects depended on the properties of the resins and the solution chemistry. Figure 8 illustrates the proposed interaction mechanism. For the resins with high S_BET_, A ensured their high adsorption efficiency although accompanied with I3 at pH 5.6. By contrast, only weak I3 occurred at pH 10. The adsorption mechanism of the resins with a high TEC was complex. A occurred constantly because of the positively charged surface. The effect of A was stronger than that of I1 and I3 at pH 5.6 but was weaker than that of I2 and I3 at pH 10, which respectively promoted and inhibited the adsorption of CPX onto the resins.

## Concusion

A series of magnetic multifunctional resins were synthesized in this study and the S_BET_ and TEC of the resins had positive correlation with the dose of DVB and GMA separately. And the effect of HA on the CPX removal by the resins was investigated.

(1) In the adsorption of CPX, hydrophobic interaction dominated for resins with high surface area and ion exchange had decisive influence for resins with high ion exchange capacity. Meanwhile, negative charge-assisted hydrogen bonds played an important role for GMA30.

(2) As competitive adsorbate, HA could directly compete and sterically shield active sites for CPX. As an adsorbent, dissociative HA in the solutions could interact with CPX to reduce CPX adsorption onto the resins. The adsorbed HA molecules onto the resins could provide additional adsorption sites for CPX and enhance CPX adsorption.

(3) At pH 5.6, the additional adsorption sites provided by adsorbed HA molecules on the resins dominated and thus facilitated the adsorption process.

(4) While at pH 10, inhibitory effects caused by HA, such as direct competition for ion exchange sites, and coexistence with CPX in the solution declined the adsorption of CPX.

## Additional Information

**How to cite this article**: Wang, W. *et al*. Effect of humic acid on ciprofloxacin removal by magnetic multifunctional resins. *Sci. Rep.* **6**, 30331; doi: 10.1038/srep30331 (2016).

## Supplementary Material

Supplementary Information

## Figures and Tables

**Table 1:** Characteristic properties of the synthesized resins.

Parameters	GMA10	GMA30	GMA50	GMA70	GMA90
GMA dose (%)	10	30	50	70	90
BET surface area (m^2^/g)	1109.25	581.19	375.72	142.87	1.71
Micropore area (m^2^/g)	205.33	120.81	55.53	9.91	1.22
Pore volume (cm^3^/g)	1.567	0.5907	0.779	0.4848	—
Micropore volume (cm^3^/g)	0.082	0.0507	0.02	0.0028	—
Mesopore volume (cm^3^/g)	1.485	0.54	0.759	0.482	—
Average pore diameter (nm)	6.49	4.76	8.37	13.57	18.5
TEC^a^ (mmol/g)	0.92	1.69	2.14	3.00	3.71
WEC^b^ (mmol/g)	0.54	1.05	1.40	2.07	2.62
SEC^c^ (mmol/g)	0.38	0.64	0.74	0.93	1.09

^a^Total anion exchange capacity.

^b^Weak anion exchange capacity.

^c^Strong anion exchange capacity.

**Table 2:** Constants for the Freundlich Langmuir and Dubinin–Radushkevich equations at 293 K in pH 5.6.

Resin	CPX	Freundlich model			Langmuir model			Dubinin-Radushkevich model		
		n	*K*_*F*_	R^2^	*K*_*L*_	Q_max_	R^2^	Q_max/100_	E	R^2^
GMA10	Single solute	1.092	1.372	0.981	2.039	0.975	0.985	1.073	7.780	0.986
	HA Competitive	1.206	1.909	0.912	4.891	0.838	0.916	0.313	10.030	0.967
	HA Preloading	1.935	0.71	0.934	21.24	0.307	0.833	0.114	12.021	0.938
GMA30	Single solute	1.084	0.655	0.983	1.425	0.628	0.984	0.482	7.823	0.993
	HA Competitive	0.988	1.364	0.971	0.523	2.647	0.971	0.591	8.100	0.979
	HA Preloading	1.303	0.667	0.938	3.034	0.496	0.911	0.137	9.901	0.949
GMA50	Single solute	1.263	0.359	0.966	4.595	0.185	0.969	0.186	8.513	0.977
	HA Competitive	1.499	0.537	0.852	11.61	0.212	0.901	0.529	8.231	0.864
	HA Preloading	1.723	0.256	0.929	15.65	0.109	0.976	0.172	8.909	0.930
GMA70	Single solute	2.046	0.139	0.858	23.21	0.064	0.935	0.069	9.872	0.901
	HA Competitive	1.418	0.537	0.809	9.499	0.215	0.848	0.141	9.872	0.946
	HA Preloading	2.299	0.193	0.898	29.61	0.094	0.908	0.046	11.578	0.958
GMA90	Single solute	1.683	0.157	0.962	13.11	0.071	0.980	0.059	9.704	0.989
	HA Competitive	1.359	0.424	0.858	8.159	0.172	0.896	0.238	8.658	0.948
	HA Preloading	1.208	1.359	0.946	2.568	0.584	0.935	0.400	8.458	0.944
