# Supplementary material for: Effect of humic acid on ciprofloxacin removal by magnetic multifunctional resins
Source: Sci Rep. 2016 Jul 28;6:30331. doi: 10.1038/srep30331 (PMC4964575; doi:10.1038/srep30331)
Supplement: Supplementary Information [file srep30331-s1.pdf]

Supplementary Information

Effect of humic acid on ciprofloxacin removal by magnetic multifunctional resins

Wei Wang, Jiade Cheng, Jing Jin, Qing Zhou*, Yan Ma, Qingqing Zhao, Aimin Li*

State Key Laboratory of Pollution Control and Resource Reuse, School of the Environment, Nanjing University, , Nanjing 210023, P. R. China

6 pages, 1 table, 5 figures

*Corresponding Author:

Qing Zhou

School of the Environment, Nanjing University,

No.163 Xianlin Avenue, Nanjing, 210023, China

Tel.: +86-25-89680377. Fax: +86-25-89680377. E-mail: zhouqing@nju.edu.cn

Aimin Li

E-mail: liaimingroup@nju.edu.cn

Synthesis of the multi-functional resins. The magnetic aminated hypercrosslinked resins were obtained via a sequence of polymerization, amination and post-crosslinking reactions[1]. For the polymerization process, 75 g of five different proportions of DVB and GMA were mixed with 7.5 g of oleic acid-coated Fe_3O_4, followed by the addition of 150 g of toluene as porogen and 1 g of benzoperoxide as initiator. The ratios of the content of GMA were from 10% to 90%. Then, the above oil phase was mixed with 500 mL of water, 4 g of gelatin, 2.1 g of trisodium phosphate, 8.4 g of disodium hydrogen phosphate and 65 g of sodium chloride in a 1 L three-necked flask and heated to 368 K for 12 h. Dimethylamine was adopted for the amination process. The obtained magnetic poly (DVB-co-GMA) beads were immersed in 250 mL of ethanol and fully swelled for 1 h. Subsequently, the mixture was heated gradually to 333 K with the addition of dimethylamine. Eventually, the post-crosslinking process was catalyzed by anhydrous ferric chloride after swelling the aminated beads in dichloromethane for 6 h. The product resin GMA10, GMA30, GMA50, GMA70 and GMA90 were rinsed repeatedly with distilled water and dried under the temperature of 333 K.

References:

1. Wang, W. et al. Two novel multi-functional magnetic adsorbents for effective removal of hydrophilic and hydrophobic nitroaromatic compounds. J. Hazard. Mater. **294,** 158-167 (2015).

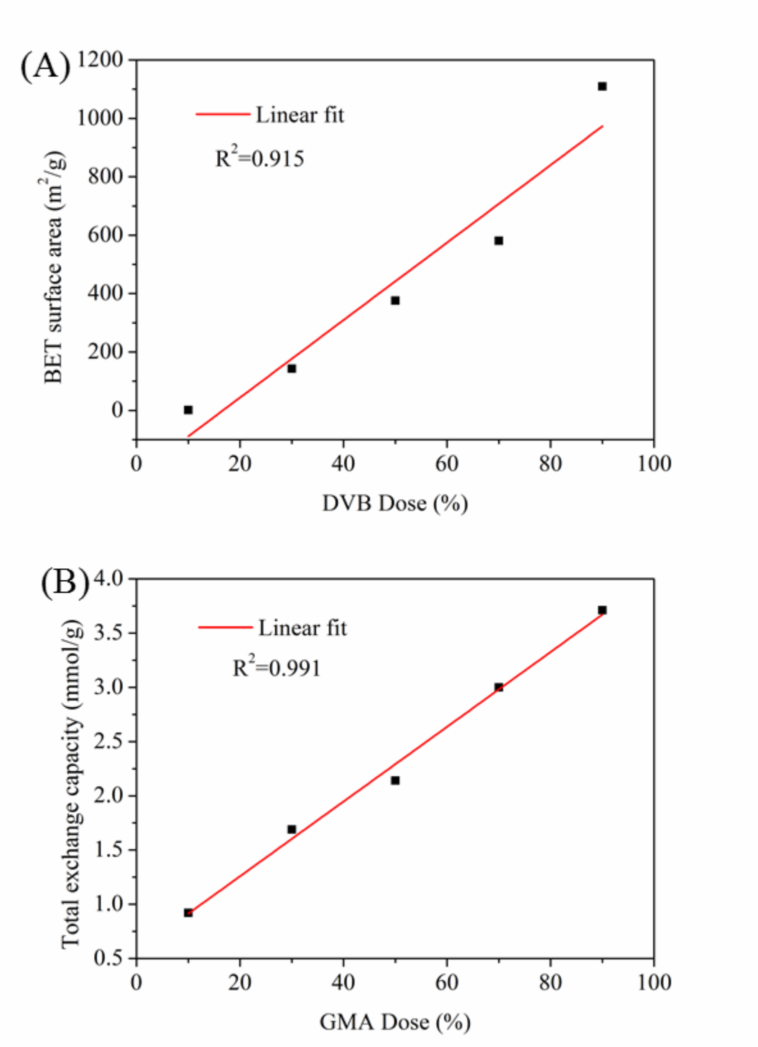

Figure S1 The correlations between (A) BET surface area and DVB Dose (B) Total exchange capacity and GMA Dose.

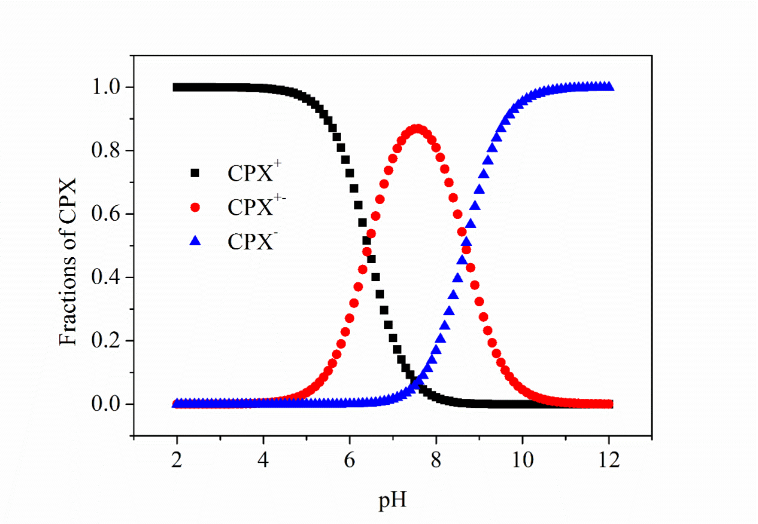

Figure S2 The fraction of cationic, amphoteric, and anionic forms of CPX at different

pH.

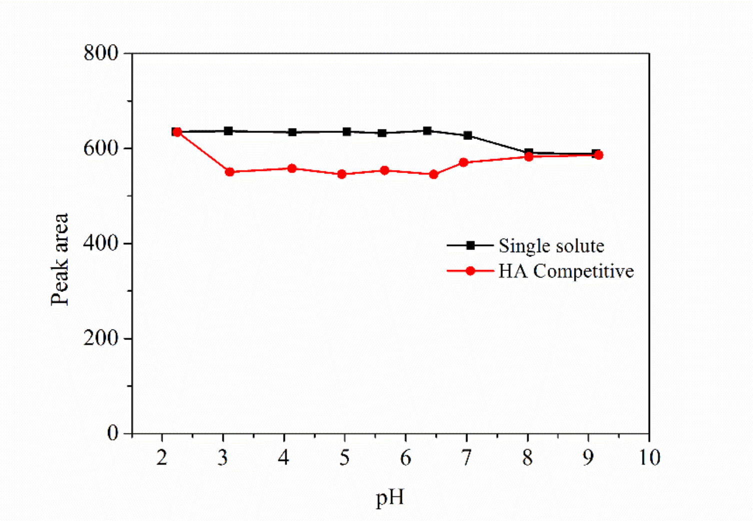

Figure S3 The peak area of CPX in single solute and HA-CPX competitive system (The concentration of CPX and HA were 0.03 mmol/L and 20 mg/L, respectively).

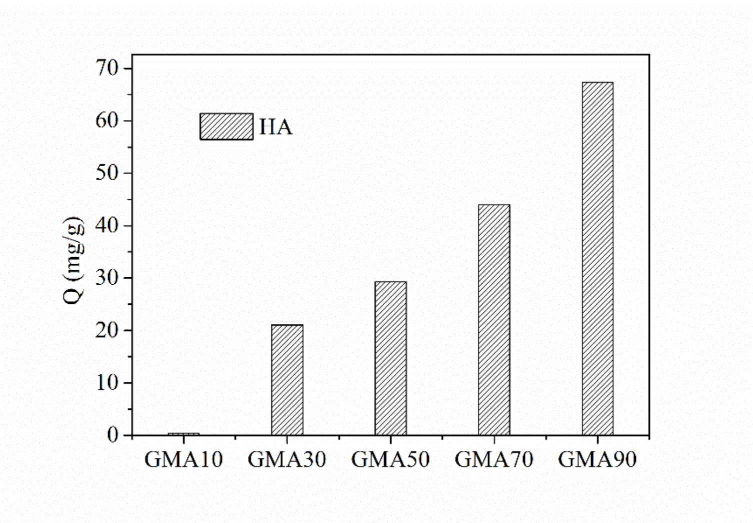

Figure S4 The adsorption amount of HA by multi-functional resins in preloading systems (C_{HA}= 20 mg/L, 0.01 g of adsorbents, temperature = 293 K).

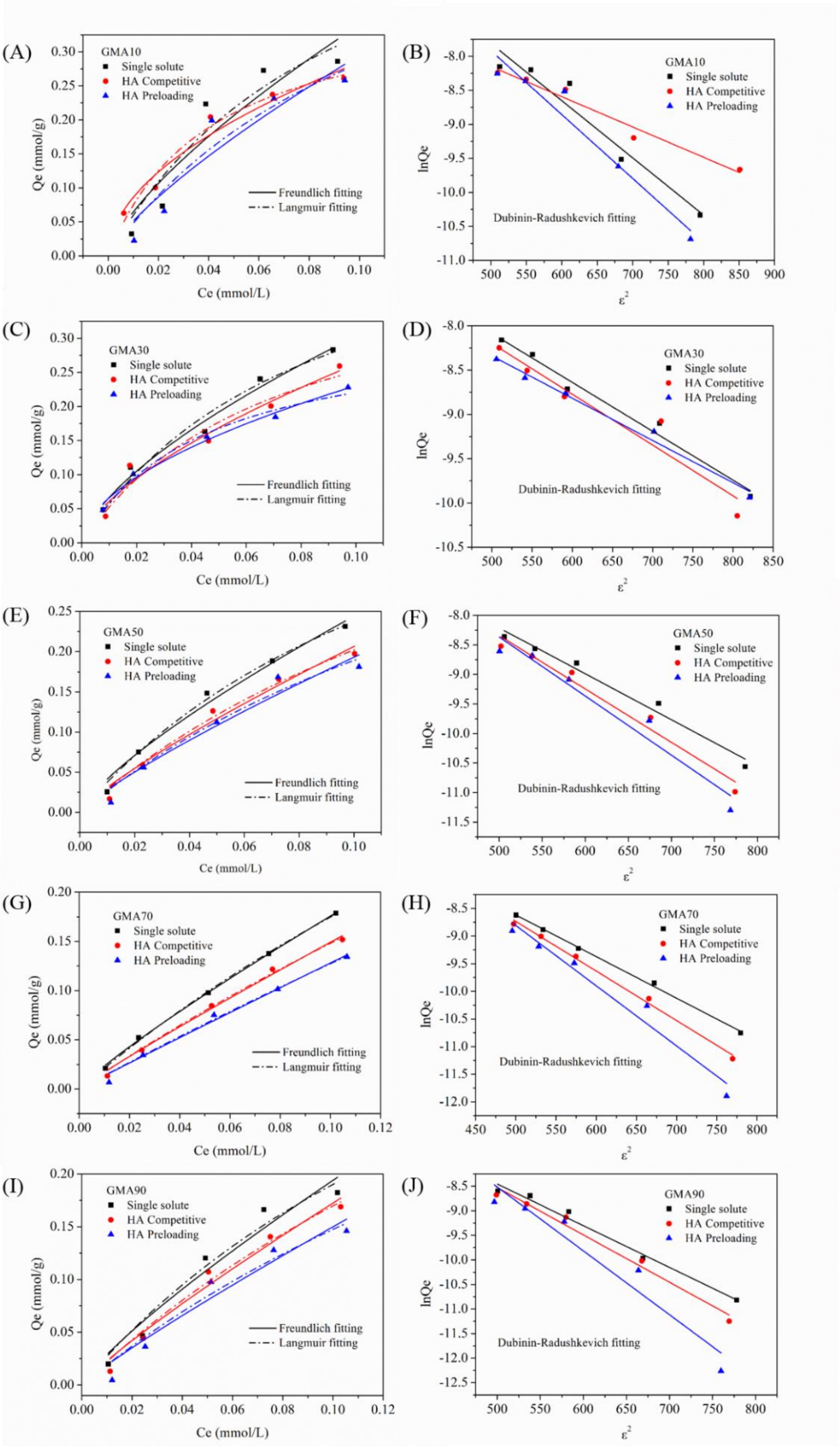

Figure S5 Adsorption isotherms of CPX onto (A, B) GMA10, (C, D) GMA30, (E, F) GMA50, (G, H) GMA70, and (I, J) GMA90 (pH=10, temperature = 293 K).

Table S1 Constants for the Freundlich Langmuir and Dubinin-Radushkevich equations at 293 K in pH 10

Resin	CPX	Freundlich model			Langmuir model			Dubinin-Radushkevich model		
		n	K_F	R^2	K_L	Q_{max}	R^2	$Q_{max/100}$	E	R^2
GMA10	Single solute	1.391	1.782	0.827	10.43	0.634	0.874	2.721	7.711	0.925
	HA Competitive	1.927	0.94	0.941	24.36	0.383	0.966	0.268	10.600	0.960
	HA Preloading	1.316	1.696	0.852	8.439	0.619	0.889	4.004	7.297	0.932
GMA30	Single solute	1.522	1.376	0.976	11.93	0.535	0.966	0.485	9.517	0.975
	HA Competitive	1.575	1.132	0.943	13.76	0.434	0.925	0.484	9.333	0.902
	HA Preloading	1.843	0.805	0.985	22.01	0.321	0.981	0.259	10.238	0.983
GMA50	Single solute	1.302	1.431	0.977	6.925	0.581	0.991	1.321	8.181	0.972
	HA Competitive	1.213	1.377	0.965	5.031	0.604	0.980	2.184	7.441	0.963
	HA Preloading	1.196	1.323	0.933	4.833	0.581	0.952	3.456	7.071	0.936
GMA70	Single solute	1.143	1.318	0.999	2.493	0.876	0.999	0.778	8.316	0.997
	HA Competitive	1.069	1.286	0.991	1.622	1.061	0.994	1.419	7.470	0.994
	HA Preloading	1.020	1.224	0.985	0.830	1.662	0.986	3.456	6.776	0.955
GMA90	Single solute	1.208	1.306	0.937	4.939	0.575	0.956	1.482	8.452	0.980
	HA Competitive	1.139	1.301	0.964	3.453	0.661	0.975	2.448	7.206	0.975
	HA Preloading	1.111	1.191	0.924	3.308	0.593	0.938	13.319	6.455	0.912
